# IL-25 Induced ROS-Mediated M2 Macrophage Polarization via AMPK-Associated Mitophagy

**DOI:** 10.3390/ijms23010003

**Published:** 2021-12-21

**Authors:** Mei-Lan Tsai, Yi-Giien Tsai, Yu-Chih Lin, Ya-Ling Hsu, Yi-Ting Chen, Ming-Kai Tsai, Wei-Ting Liao, Yi-Ching Lin, Chih-Hsing Hung

**Affiliations:** 1 Graduate Institute of Medicine, College of Medicine, Kaohsiung Medical University, Kaohsiung 807, Taiwan; snoopy905@gmail.com (M.-L.T.); yainghsu@kmu.edu.tw (Y.-L.H.); 2 Department of Pediatrics, Faculty of Pediatrics, College of Medicine, Kaohsiung Medical University, Kaohsiung 807, Taiwan; pinkkitty1121@gmail.com; 3 Department of Pediatrics, Changhua Christian Children Hospital, Changhua 500, Taiwan; 107239@cch.org.tw; 4 School of Medicine, Kaohsiung Medical University, Kaohsiung 807, Taiwan; 5 School of Medicine, Chung Shan Medical University, Taichung 402, Taiwan; 6 Department of Medical Humanities and Education, School of Medicine, Kaohsiung Medical University, Kaohsiung 807, Taiwan; springfred@gmail.com; 7 Department of Internal Medicine, Division of Allergology, Immunology and Rheumatology, Kaohsiung Medical University, Kaohsiung 807, Taiwan; 8 Drug Development and Value Creation Research Center, Kaohsiung Medical University, Kaohsiung 807, Taiwan; 9 Department of Internal Medicine, Division of Nephrology, Kaohsiung Armed Forces General Hospital, Kaohsiung 802, Taiwan; tmk802@gmail.com; 10 Department of Medical Research, Kaohsiung Medical University Hospital, Kaohsiung Medical University, Kaohsiung 807, Taiwan; 11 Department of Biotechnology, College of Life Science, Kaohsiung Medical University, Kaohsiung 807, Taiwan; 12 Department of Laboratory Medicine, Kaohsiung Medical University Hospital, Kaohsiung Medical University, Kaohsiung 807, Taiwan; 13 Doctoral Degree Program of Toxicology, College of Pharmacy, Kaohsiung Medical University, Kaohsiung 807, Taiwan; 14 Department of Laboratory Medicine, School of Medicine, College of Medicine, Kaohsiung Medical University, Kaohsiung 807, Taiwan; 15 Department of Pediatrics, Kaohsiung Medical University Hospital, Kaohsiung Medical University, Kaohsiung 807, Taiwan; 16 Research Center for Environmental Medicine, Kaohsiung Medical University, Kaohsiung 807, Taiwan; 17 Department of Pediatrics, Kaohsiung Municipal Siaogang Hospital, Kaohsiung 812, Taiwan

**Keywords:** IL-25, reactive oxygen species, AMPK, mitophagy, M2 macrophage polarization

## Abstract

Interleukin (IL)-25 is a cytokine released by airway epithelial cells responding to pathogens. Excessive production of reactive oxygen species (ROS) leads to airway inflammation and remodeling in asthma. Mitochondria are the major source of ROS. After stress, defective mitochondria often undergo selective degradation, known as mitophagy. In this study, we examined the effects of IL-25 on ROS production and mitophagy and investigated the underlying mechanisms. The human monocyte cell line was pretreated with IL-25 at different time points. ROS production was measured by flow cytometry. The involvement of mitochondrial activity in the effects of IL-25 on ROS production and subsequent mitophagy was evaluated by enzyme-linked immunosorbent assay, Western blotting, and confocal microscopy. IL-25 stimulation alone induced ROS production and was suppressed by N-acetylcysteine, vitamin C, antimycin A, and MitoTEMPO. The activity of mitochondrial complex I and complex II/III and the levels of p-AMPK and the mitophagy-related proteins were increased by IL-25 stimulation. The CCL-22 secretion was increased by IL-25 stimulation and suppressed by mitophagy inhibitor treatment and PINK1 knockdown. The Th2-like cytokine IL-25 can induce ROS production, increase mitochondrial respiratory chain complex activity, subsequently activate AMPK, and induce mitophagy to stimulate M2 macrophage polarization in monocytes.

## 1. Introduction

Interleukin (IL)-25 is an epithelial-derived cytokine that links innate and adaptive immunity by enhancing Th2 responses [1,2]. IL-25 is produced by immune cells and epithelial cells [3]. It regulates the internal safety of adaptive immune responses, leading to the initiation of allergic diseases and playing a role in stimulating of pulmonary mucosal cells and fibroblasts [1,4]. IL-25 production in humans and mice or injection of IL-25 into animals has been shown to result in the secretion of high levels of Th2 cytokines, such as IL-4, IL-5, and IL-13 [5]. Pilot studies have shown that IL-25 mRNA is highly expressed in Th2 cells. Furthermore, in a murine pulmonary fibrosis model, collagen deposition in the lungs of challenged mice was driven by IL-25-induced type 2 innate lymphoid cells (ILC2s)-released IL-13 [6]. Therefore, IL-25 and ILC2s could be therapeutic targets of human fibrotic diseases.

The physiological damage caused by oxidative stress resulting from reactive oxygen species (ROS) has been demonstrated to induce airway hyperresponsiveness (AHR) by smooth muscle contraction and enhance mucus secretion and epithelial shedding [7]. Increased generation of ROS has been demonstrated in patients with asthma. Overexpression of ROS leads to oxidative stress, contributing to the emergence and persistence of pulmonary fibrosis induced by TGF-β. ROS are considered to be vital mediators of pulmonary vascular cell proliferation and enhance VEGF expression, which increases the thickness of pulmonary arterial walls and promotes vascular remodeling [8].

Mitochondria-specific autophagy is called mitophagy and occurs in defective mitochondria after damage or stress. The dysregulation of mitophagy plays an important role in the development of chronic diseases, including neurodegenerative diseases, metabolic diseases, and heart failure [9]. PTEN-induced putative kinase 1 (PINK1) is a serine/threonine kinase that contains a mitochondrial targeting sequence, and Parkin is required for mitophagy induction. The pathways of mitophagy initiation overlap considerably with those required for general autophagy [10] and have been shown to contribute to pulmonary fibrosis [11]. Thus, ROS production in structural lung cells, such as epithelial cells, is also likely to play an important role [12]. IL-25 is a Th2-like cytokine, and several studies have suggested its relationship with asthma [13]. However, the detailed mechanisms underlying the effect of IL-25 on ROS production remain unclear. In the present study, we investigated the effects of IL-25 on ROS production and the possible underlying mechanisms in human monocytes.

## 2. Results

The Th2-like cytokine IL-25 induced intracellular ROS production in THP-1 cells and THP-1 derived macrophages.

**Figure 1:**
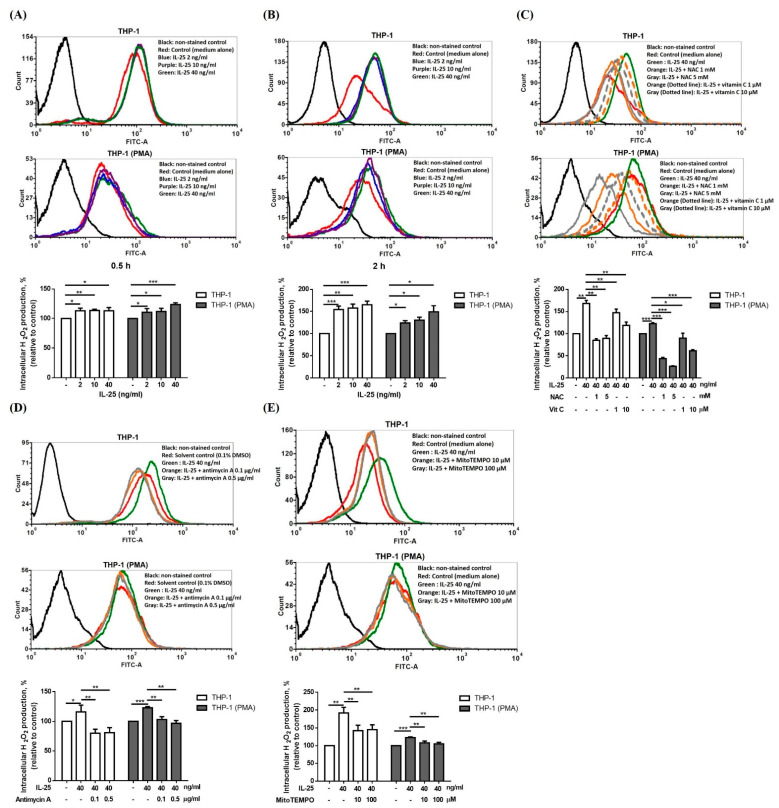
IL-25 alone could induce intracellular ROS level and suppressed by NAC, vitamin C, antimycin A and MitoTEMPO. (**A**–**E**), representative histograms of cell counts (counts) vs. DCF fluorescence (FITC-A) (above), and the mean fluorescence intensity (MFI) of DCF is expressed as percentage relative to control cells (below). THP-1 cells (white bar) and THP-1 derived macrophages (gray bar) treated with medium alone or IL-25 (2, 10 and 40 ng/mL) was for 0.5 h (**A**) or 2 h (**B**). (**C**) Cells were treated with medium alone or IL-25 (40 ng/mL) present pretreatment with NAC (1 and 5 mM) or vitamin C (1 and 10 μM) for 0.5 h. After pretreatment with solvent control (0.1% DMSO) or antimycin A (0.1 and 0.5 μg/mL) (**D**), or medium alone or MitoTEMPO (10 and 100 μM) (**E**) for 0.5 h followed by IL-25 treatment, the ROS level was measurement. * *p* < 0.05, ** *p* < 0.01, and *** *p* < 0.001; n = 3, means ± SD.

**Figure 2:**
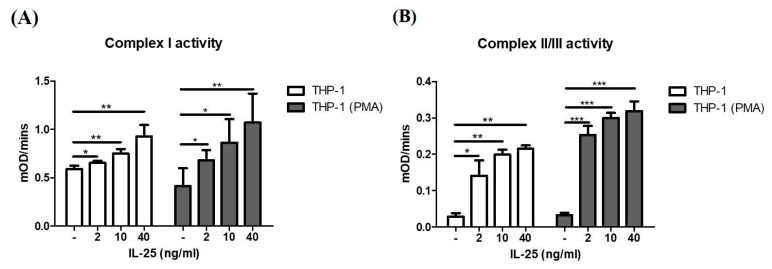
IL-25 increased mitochondria complex activity in THP-1 cells and THP-1 derived macrophages. THP-1 cells (white bar) and THP-1 derived macrophages (gray bar) were treated with medium alone or IL-25 (2, 10, and 40 ng/mL) for 2 h. The activity of complex I (**A**) and complex II/III (**B**) by IL-25 treatment were determined. * *p* < 0.05, ** *p* < 0.01, and *** *p* < 0.001; n = 4, means ± SD.

IL-25 is defined as an upstream epithelial cytokine driving the production of potent Th2 cytokines, such as IL-5 and IL-13, which have been proven to be involved in severe, difficult-to-treat eosinophilic asthma. Therefore, we first examined the effect of another Th2-like cytokine, IL-25, on ROS production in THP-1 cells and THP-1 derived macrophages. IL-25 (2–40 ng/mL) induced ROS production in THP-1 cells and THP-1 derived macrophages at the 0.5 h (Figure 1A) and 2 h (Figure 1B) time points. We selected 2 h as a detection time point to investigate the antioxidative effect on IL-25-induced ROS production. The IL-25-induced ROS production in THP-1 cells and THP-1 derived macrophages were significantly inhibited by NAC, vitamin C (Figure 1C). Moreover, we further used mitochondrial complex III inhibitor antimycin A and mitochondrial-targeting antioxidant MitoTEMPO to examine the mitochondria-specific antioxidative effect. The IL-25-induced ROS production in THP-1 cells and THP-1 derived macrophages were also significantly suppressed by antimycin A (Figure 1D) and MitoTEMPO (Figure 1E).

### 2.1. IL-25 Increased Mitochondrial Complex I and II/III Activity

IL-25 could induce ROS production in THP-1 cells and THP-1 derived macrophages, and mitochondria are the major source of ROS production. Therefore, we further measured the mitochondrial complex activity of THP-1 cells and THP-1 derived macrophages after IL-25 stimulation. As shown in Figure 2A, the activity of mitochondrial complex I in THP-1 cells and THP-1 derived macrophages was significantly increased by IL-25 stimulation in a dose-dependent manner. In addition, we also observed that IL-25-induced mitochondrial complex II/III activity of THP-1 cells and THP-1 derived macrophages was significantly increased (Figure 2B).

### 2.2. IL-25 Induced AMPK Activation and Mitophagy-Related Proteins Expression

**Figure 3:**
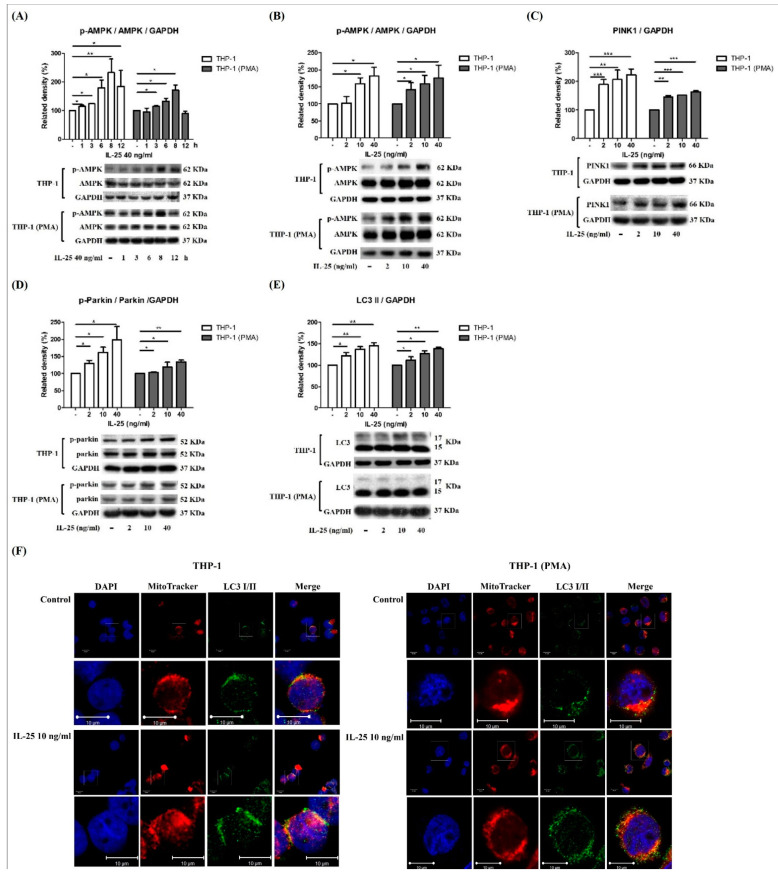
IL-25-induced AMPK activation and mitophagy-related proteins expression in THP-1 cells and THP-1 derived macrophages. (**A**) The time course of p-AMPK expression was determined at 1, 3, 6, 8, and 12 h with IL-25 (40 ng/mL) or medium alone treatment in THP-1 cells (white bar) and THP-1 derived macrophages (gray bar). Data present the means ± SD of 3 independent experiments. The expression of p-AMPK (**B**), PINK1 (**C**), p-parkin (**D**), and LC3 (**E**) by medium alone or IL-25 (2, 10, and 40 ng/mL) treatment in THP-1 cells (white bar) and THP-1 derived macrophages (gray bar) at 8 h time point were determined by western blot. * *p* < 0.05, ** *p* < 0.01, and *** *p* < 0.001; n = 3, means ± SD. The confocal data show the nuclear (DAPI), mitochondria (MitoTracker), and LC3 I/II fluorescence images by medium alone or IL-25 (10 ng/mL) treatment (**F**). Scale bars = 10 μm in pictures.

IL-25 significantly induces ROS production, and ROS plays an important role in mitophagy. Mitophagy is essential for cellular energy and maintaining redox homeostasis and important for mitochondrial quality control [14]. Previous research observed that a lack of skeletal muscle AMPK resulted in the failed induction of mitophagy-related signaling and development of apparent mitochondrial abnormalities. [15]. Thus, we further examined whether IL-25 can also induce AMPK activation and mitophagy-related proteins expression in THP-1 cells and THP-1 derived macrophages. Western blot analysis showed that IL-25 significantly increased the levels of p-AMPK at 12 h in THP-1 cells but not in THP-1 derived macrophages. However, the best time point of p-AMPK activation is 8 h (Figure 3A). In THP-1 cells and THP-1 derived macrophages, the expression of p-AMPK and the mitophagy-related proteins PINK1, p-Parkin, and LC3 was significantly increased by IL-25 treatment with a dose-dependent manner at 8 h (Figure 3B–E). Costaining of mitochondria and LC3 was used to monitor IL-25-induced mitophagy. As shown in Figure 3F, mitochondria and LC3 were colocalized, and their colocalization increased in response to 10 ng/mL IL-25 stimulation in THP-1 cells and THP-1 derived macrophages, as shown by confocal laser microscopy analysis.

### 2.3. IL-25 Induced Mitophagy through the ROS-AMPK Pathway

AMPK has been suggested to be a redox-sensing protein that mediates mitophagy-related signaling.

**Figure 4:**
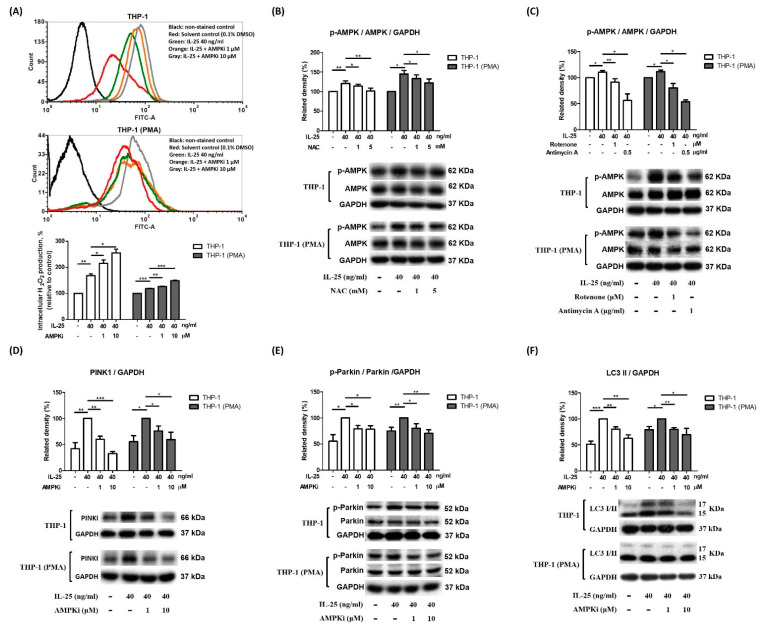
IL-25-induced mitophagy-related proteins expression via ROS-AMPK pathway in THP-1 cells and THP-1 derived macrophages. (**A**) representative histograms of cell counts (counts) vs. DCF fluorescence (FITC-A) (above), and the mean fluorescence intensity (MFI) of DCF expressed as a percentage relative to control cells (below). (**A**) After pretreatment with solvent control (0.1% DMSO) or AMPKi (1 and 10 µM) for 0.5 h, the IL-25-induced ROS production in THP-1 cells (white bar) and THP-1 derived macrophages (gray bar) were measured. n = 3, means ± SD. (**B**) After medium alone or NAC (1 and 5 mM) pretreatment for 0.5 h followed by IL-25 treatment for 8 h, IL-25-induced p-AMPK expression in THP-1 cells (white bar) and THP-1 derived macrophages (gray bar) were determined. n = 4, means ± SD. (**C**) After pretreatment with solvent control (0.1% DMSO), rotenone (1 μM) or antimycin A (0.5 μg/mL) for 0.5 h followed by IL-25 treatment for 8 h, IL-25-induced p-AMPK expression in THP-1 cells (white bar) and THP-1 derived macrophages (gray bar) were deter-mined. n = 3, means ± SD. After pretreatment with solvent control (0.1% DMSO) or AMPKi (1 and 10 µM) for 0.5 h followed by IL-25 treatment for 8 h, the PINK1 (**D**), p-Parkin (**E**), and LC3 I/II (**F**) protein levels were determined by western blot. n = 3, means ± SD. * *p* < 0.05, ** *p* < 0.01, and *** *p* < 0.001.

**Figure 5:**
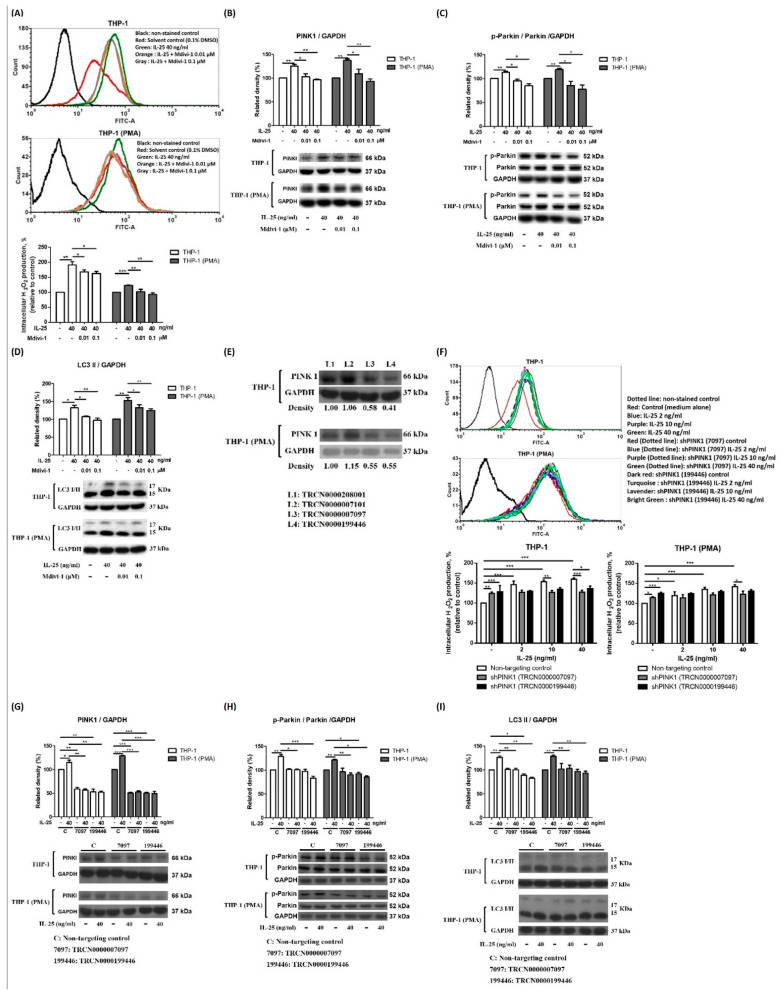
IL-25-induced mitophagy via PINK1-Parkin pathway in THP-1 cells and THP-1 derived macrophages. (**A**,**F**) representative histograms of cell counts (counts) vs. DCF fluorescence (FITC-A) (above), and the mean fluorescence intensity (MFI) of DCF expressed as a percentage relative to control cells (below). (**A**) After pretreat-ment with solvent control (0.1% DMSO) or mdivi-1 for 0.5 h, the IL-25-induced ROS production in THP-1 cells (white bar) and THP-1 derived macrophages (gray bar) were measured by flow cytometry. n = 3, means ± SD. After pretreatment with solvent control (0.1% DMSO) or mdivi-1 for 0.5 h followed by IL-25 treatment for 8 h, the PINK1 (**B**), p-Parkin (**C**), and LC3 I/II (**D**) protein levels of THP-1 cells (white bar) and THP-1 derived macrophages (gray bar) were determined. n = 3, means ± SD. (**E**) After transduction with shRNA lentiviral particles and puromycin selection for 3 d, the PINK1 expression was determined. (**F**) After PINK1 knockdown by TRCN0000007097 or TRCN0000199446 shRNA, the ROS level by medium alone or IL-25 (2, 10, and 40 ng/mL) treatment in THP-1 cells at 2 h was measured. n = 3, means ± SD. The expression of PINK1 (**G**), p-Parkin (**H**), LC3 I/II (**I**) by medium alone or IL-25 (40 ng/mL) treatment following transduction with PINK1 shRNA or non-targeting control were determined. n = 3, means ± SD. * *p* < 0.05, ** *p* < 0.01, and *** *p* < 0.001.

Therefore, we further examined the relationship between ROS and AMPK activation after IL-25 stimulation. In this study, pretreated AMPKi caused enhanced ROS production in IL-25-treated THP-1 cells and THP-1 derived macrophages (Figure 4A). Moreover, we also used NAC, rotenone, and antimycin A to observe whether IL-25 induced AMPK activation. The IL-25-increased p-AMPK expression was suppressed by NAC (Figure 4B). The IL-25-increased p-AMPK was also significantly suppressed by rotenone and antimycin A (Figure 4C). Thus, it suggested that complexes I and III produced mitochondrial ROS involved in AMPK activation. We further used the AMPKi to examine whether IL-25 induced mitophagy-related proteins expression in THP-1 cells and THP-1 derived macrophages. The Western blot data showed that IL-25-induced PINK expression was suppressed by treatment with the AMPKi (Figure 4D). In addition, the IL-25-induced increases in the levels of p-Parkin and LC3 were similarly suppressed by treatment with the AMPKi (Figure 4E,F). The data supported that L-25-induced mitophagy was through increased ROS production and AMPK activation. We further used mdivi-1 to investigate ROS production and mitophagy-related proteins expression on IL-25 treatment in THP-1 cells and THP-1 derived macrophages. As shown in Figure 5A, IL-25-induced ROS production was inhibited by mdivi-1 pretreatment. The IL-25-induced mitophagy-related protein (PINK1, p-Parkin, and LC3) expression decreased by mdivi-1 (Figure 5B–D). Furthermore, we also knock down PINK1 to examine whether IL-25 induced ROS production and mitophagy-related proteins expression. The knockdown efficiency of PINK1 is shown in Figure 5E. We selected TRCN0000007097 and TRCN0000199446 shRNA to investigate further experiments. After PINK1 knockdown, the ROS production of THP-1 cells and THP-1 derived macrophages was increased without IL-25 stimulation. Moreover, ROS production was not enhanced by IL-25 treatment (Figure 5F). The data supported that PINK1 was involved in IL-25-induced ROS production. We further examine whether IL-25 induced mitophagy-related protein expression in THP-1 cells and THP-1 derived macrophages. As shown in Figure 5G, the result indicated the successful knockdown PINK1. After PINK1 knockdown, the PINK1 downstream proteins (p-Parkin and LC3) were not affected by IL-25 treatment (Figure 5H,I). Together, these results suggested that IL-25-induced mitophagy via PINK1-Parkin pathway through ROS and AMPK activation.

### 2.4. IL-25-Induced Mitophagy Was Associated with an M2 Macrophage Polarization Shift

**Figure 6:**
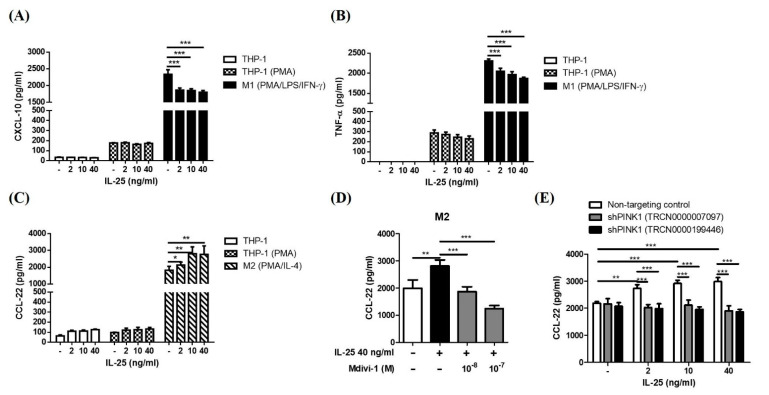
IL-25-induced mitophagy was associated with a shift in M2 macrophage polarization. THP-1 cells were pretreated with medium alone or IL-25 (2, 10, and 40 ng/mL) for 2 h and differentiated to THP-1 derived macrophages (PMA), M1 (PMA/LPS/IFN-γ), or M2 (PMA/IL-4) macrophages. The concentration of M1-related chemokine CXCL-10 (**A**) and cy-tokine TNF-α (**B**) and M2-related cytokine CCL-22 (**C**) were measured. After pretreatment with solvent control (0.1% DMSO) or mdivi-1 (**D**), or knockdown PINK1 (**E**), the IL-25-induced CCL-22 production was assessed. * *p* < 0.05, ** *p* < 0.01, and *** *p* < 0.001; n = 3, means ± SD.

To evaluate the functional outcomes of IL-25-induced mitophagy in THP-1 cells, we examined the effects of IL-25 on macrophage polarization. IL-25 stimulation significantly decreased the release of the M1-related cytokines CXCL-10 and TNF-α in THP-1-derived M1 cells but did not show the effect from THP-1 cells and THP-1-derived macrophage. (Figure 6A,B). In contrast, the M2-related cytokine CCL-22 was significantly increased in THP-1-derived M2 cells but not shown in THP-1 cells and THP-1- derived macrophage (Figure 6C). These results suggested that IL-25 promoted macrophage polarization toward the M2 phenotype. Furthermore, IL-25-induced production of M2-related cytokines was suppressed by treatment with the Mdivi-1 or genetic knockdown of PINK1 (Figure 6D,E), indicating that the IL-25-induced M2 macrophage polarization shift was mediated by mitophagy.

## 3. Discussion

Asthma is a chronic disease characterized by reversible airflow limitation, bronchial constriction, and inflammatory immune response. Epithelial cells are the first line against allergen exposures and environmental stimuli via the expression of many cytokines and chemokines such as IL-25. IL-25 induces proallergic chemokine production, an increase in goblet cells, and mucus secretion in airway epithelium, resulting in epithelial hyperplasia and airway hyperreactivity [16]. IL-25 also mediated immune cells, such as dendritic cells (DCs) and macrophages, to activate a type 2 immune response. A previous study demonstrated the IL-25 receptor IL-17RB expression of memory Th2 cells was significantly increased by coculture with TSLP-conditioned DCs (CD11c^+^ DCs cultured with 15 ng/mL of TSLP for 24 h, then washout) and enhanced proliferation of memory Th2 cells by IL-25 stimulation [17]. In a human study, the IL-17RB was upregulated in myeloid DCs (mDCs) and plasmacytoid DCs via allergen challenge and coincident with increases in airway eosinophils. Thus, it suggested that IL-25 may activate mDCs to initiate type 2 immune responses [18]. In the airway, macrophages are the most abundant immune cells and interact with epithelial cells to maintain immune tolerance and tissue repair [19]. However, few studies have investigated epithelial–macrophage crosstalk, even the role of IL-25 in macrophages. ROS are involved in many cell physiological processes. However, they are also implicated in the pathogenesis of tissue injury. In the asthmatic airway, inflammatory cells are recruited and produce various highly reactive ROS to promote tissue damage and chronic airway inflammation [20,21]. The main sources of oxidants in the bronchial airways are environmental pollution, e.g., particulate matter (PM) and endogenously produced oxidants due to local inflammation [22]. IL-25, also named IL-17E, is a distinct member of the IL-17 cytokine family, whose members can promote and augment Th2 cells. Clinical studies have indicated that IL-25 production is associated with eosinophilic inflammation and mucus hypersecretion, and with AHR and airway remodeling [13]. In the airway, the direct targets of inhaled PM are epithelial cells and antigen-presenting dendritic cells (DCs). Several cytokines are mediated by epithelial cells, including IL-25, which promoting the DC response to allergens and PM. PM components are also redox-active and can induce cellular oxidative stress and injuries, including inflammation and cell death [23]. Therefore, recent studies have suggested that a novel method to monitor disease severity is to assess oxidative stress byproducts. In addition, a potential treatment strategy was used in asthmatic patients by reducing ROS levels to decrease airway inflammation, which is produced by oxidative stress [24]. The present study results showed that IL-25 could increase ROS production in monocytes, suggesting that IL-25 may be important not only in the induction of Th2 cytokine production, but also as a potent source of oxidative stress. In our data, the intracellular ROS level was not obviously different between THP-1 and THP-1 (PMA) cells. Both monocytes and macrophages could produce ROS. However, macrophages have more oxidative burst activity and reactive oxygen species (ROS) production due to their capacity to phagocytize foreign materials, especially M1 macrophage [25]. Our experiment condition only added PMA, not combined IFN-γ and LPS, and differentiated 24 h, not three days or more. Our PMA-primed THP-1 cells are not mature M1 macrophages. The production and release of epithelial cytokines, including IL-33, IL-25, and TSLP, lead to the recruitment and activation of ILC2, which secretes mediators, such as IL-5 and IL-13, that augment allergic inflammation in the pathogenesis of virus-induced asthma exacerbation. The most common cause of virus-induced asthma exacerbation and accounting for much of the morbidity burden of asthma is rhinovirus (RV) infection. RV-infected asthmatic bronchial epithelial cells displayed an enhanced intrinsic capacity for IL-25 expression, which could contribute to asthma exacerbation [26]. Early-life human RV infection has been associated with asthma development in high-risk infants and children. An RV-induced asthma animal model revealed an increased intrinsic capacity for type 2 cytokines in the lung, and IL-25 is a key mediator of RV-induced exacerbation of pulmonary inflammation, especially in early life [27]. It has been reported that the levels of IL-25 and its receptor IL-17RB were increased in respiratory syncytial virus (RSV) infection, and RSV-associated AHR and type 2 cytokine production were reduced because of IL-25 blockade by neutralizing antibodies. An IL-17RB^−/−^ mouse study demonstrated that decreases in Th2 cytokines and increases in Th17 cytokines reduced inflammation in mice with RSV-induced asthma exacerbation [28]. Therefore, the RSV-induced inflammatory response was regulated by IL-25, which might reduce the severity of RSV-associated pulmonary inflammation and viral-induced asthma exacerbation via IL-25 blockade. These environmental factors increase the levels of cellular ROS and induce mitochondrial dysfunction in the airway epithelium of asthmatic patients [29]. The imbalance in mitochondrial ROS production, which consists principally of the generation of superoxide and hydrogen peroxide, has been implicated in dysfunction in the electron transport chain (ETC), resulting in altered ROS production. However, the response of IL-25, a Th2-like cytokine, to the ETC has not been thoroughly assessed. Complex I and III redox centers in the respiratory chain of mitochondria have been implicated as the major sites of mitochondrial ROS production, and complex II is also capable of producing ROS [30,31]. In the present study, our results showed that the complex I and II/III activities after 2 h of stimulation by IL-25 were significantly increased compared to the control group. Thus, IL-25 may increase ROS generation by expanding complex I and II/III activity. However, an increase in the activity of the complexes is not necessarily related to a rise in ROS levels. Previous research showed increased ROS production but reduced complex I activity in mitochondria from reperfused rat heart [32]. Other research indicated that mitochondria’s complex II or III activity increase could follow increased complex II or III-associated ROS production [33,34]. The present study showed IL-25 could increase mitochondrial complex I and II/III activity and ROS production.

Mitochondria play an important metabolic role, cause cellular damage via oxidative phosphorylation byproducts, and trigger apoptosis via cytochrome c release from damaged mitochondria and the activation of cell death pathways [35]. Mitophagy is the selective degradation of mitochondria by autophagy and occurs in defective mitochondria following damage or stress. The pathways for generating mitophagy have been identified and are primarily mediated by PINK1 and the E3 ubiquitin ligase Parkin [36,37]. PINK1 is constitutively expressed and imported to healthy mitochondria via the translocase TIM/TOM complex to the inner membrane and is cleaved by presenilin-associated rhomboid-like protein (PARL). Parkin is kept in a ‘closed’ confirmation through multiple intramolecular interactions that keep the enzyme in an autoinhibited state. In damaged mitochondria, activated PINK1 accumulates on the mitochondrial surface upon loss of mitochondrial membrane potential [38]. A previous study indicated that PINK1 could be autophosphorylated by PINK kinase. Accumulated PINK1 activates Parkin by phosphorylating the Ser-65 residue of Parkin and recruiting Parkin to mitochondria to trigger mitophagy [39]. Recently, the mechanism of mitochondrial dysfunction has been established to be linked with the pathogenesis of chronic respiratory diseases by playing a causative role in the structural remodeling of the lung. TGF-β1, a key cytokine for asthma-related pulmonary fibrosis, could induce lung epithelial cell mitochondrial ROS and stabilize the key mitophagy initiating protein PINK1. PINK1 ameliorates epithelial cell death and is associated with fibrogenesis [40]. Increased pulmonary expression of IL-25 is found in idiopathic pulmonary fibrosis, and a population of ILC2s is also observed in the lungs of idiopathic pulmonary fibrosis patients [6]. In the present study, IL-25 induced ROS production and subsequently increased mitophagy-related PINK1, Parkin, and LC3 expression. These results may explain the association between IL-25 and idiopathic pulmonary fibrosis.

**Figure 7:**
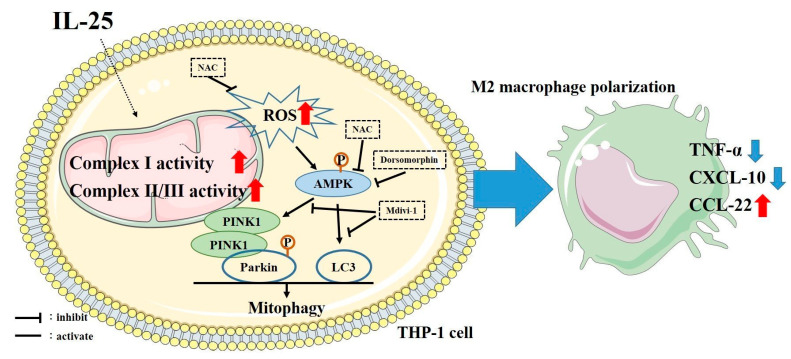
Schematic of the proposed intracellular mechanisms underlying IL-25-induced M2 macrophage polarization. IL-25 increased ROS production and mitochondrial complex I and complex II/III activity. Subsequently, activation of mitophagy, and then shifted M1/M2 chemokine expression via the AMPK signaling pathway in human monocyte cell line.

AMPK, a trimeric complex including the α, β, and γ subunits, is the main cellular energy sensor that restores energy homeostasis and coordinates metabolism. Full activation of AMPK occurs through threonine phosphorylation in the activation loop of the kinase domain, which is situated at the N-terminus of the α subunit. Increasing evidence suggests that AMPK might also be a redox-sensing protein [41]. Mitochondrial ROS activates AMPK under both resting and stress conditions [42]. Mitochondrial complexes I and III are major sources of mitochondrial ROS. In 2004, Zou MH. et al. reported that mitochondrial O_2_^●^^−^ was increased by rotenone treatment and led to increased phosphorylation of AMPK [43]. In a previous study, Rabinovitch et al. also indicated that “mitochondrial ROS are a noncanonical activator of AMPK” [44]. It seems that mitochondrial ROS produced by complexes I and III are involved in AMPK activation. A previous study observed that a nonprescription oral drug, berberine, which is adjunctively for metabolic disorders, increased peroxynitrite, a potent oxidant producer, and phospho-AMPK expression. Berberine-induced phospho-AMPK expression was attenuated by antioxidant NAC pretreatment [45]. This study also showed that phospho-AMPK expression was increased by IL-25 stimulation and was decreased by NAC pretreatment. AMPK regulates a variety of metabolic processes, including mitochondrial biology and homeostasis. In mitochondrial homeostasis, AMPK can directly phosphorylate mitochondrial fission factor (MFF) to regulate mitochondrial fission, which is required for mitophagy, and activate ULK1, the upstream kinase in autophagy and mitophagy [46]. In the present study, we used an AMPK inhibitor to investigate the effect of AMPK on regulating mitophagy-related proteins expression. Our results showed that IL-25-increased mitophagy-related proteins, as PINK1, p-Parkin, and LC3 expression were attenuated by AMPK inhibitor pretreatment. Mitochondrial biogenesis has been indicated to be regulated by peroxisome proliferator-activated receptor)-γ coactivator-1α (PGC-1α). PGC-1α has been suggested to increase the expression of key mitochondrial enzymes, such as ATP synthetase (β-subunit) and cytochrome c oxidase (COX) subunits (COX II and COX IV). An animal model showed a strong positive correlation between PGC-1α protein expression and COX activity [47]. More signalling pathways were demonstrated leading to the PGC-1α transcription cascade activation, such as p38 mitogen-activated protein kinases (p38 MAPK) and AMPK [48]. IL-25 has been shown to be mediated by their receptors IL-17RA and IL-17RB and to activate several downstream signalling cascades, including MAPKs, nuclear factor kappa B (NF-kB), and Janus kinase/signal transducer and activator of transcription (JAK/STAT) [16]. We found IL-25 could induce AMPK activation by ROS and mediated mitophagy. Taken together, it seems that IL-25 stimulated mitochondrial activity through AMPK and subsequent mitophagy. All our data also suggested that epithelium-derived IL-25 may not only induce Th2-related cytokine expression, but also ROS production, and subsequently influence cellular oxidative stress and the normal redox state and regulate mitochondrial quantity in monocytes (Figure 7). The present study indicated that IL-25 could increase intracellular ROS level and mitochondrial complex I and II/III activity and induce mitophagy through AMPK activation and PINK1-Parkin pathway in human monocyte cell line THP-1 and THP-1 (PMA). We further investigated whether IL-25 could enhance M2 chemokine CCL-22 production in M2 macrophage and suppress by mitophagy inhibitor or PINK1 knockdown. In addition, a previous study observed that mitochondrial respiratory capacity, Arg-1 (one of M2 marker), and Th2 cytokines such as IL-4, IL-5, and IL-13 were increased in IL-25-induced bone marrow-derived macrophages. Furthermore, in IL-25-treated high-fat diet-fed mice, the ARG1 and F4/80 double-positive cells accumulated in both the liver and epididymal white adipose tissue [49]. Taken together, it seems that IL-25 could train macrophages to skew towards M2 phenotype through mitophagy and induce Th2-type immune responses. Understanding the mechanisms of IL-25-induced ROS production and mitophagy, which are important factors for the pathogenesis of asthma, allergies, and other airway diseases, can help us identify viable points for therapeutic intervention in respiratory diseases and for future avenues of research in this rapidly evolving field.

## 4. Materials and Methods

### 4.1. Cell Culture

The THP-1 human monocyte cell line (American Type Culture Collection, Rockville, MD, USA) was cultured in RPMI-1640 medium containing 10% fetal bovine serum and antibiotic-antimycotic (Gibco, Carlsbad, CA, USA, #15240-062) in a humidified incubator at 37 °C and 5% CO_2_. The cells were cultured with 20 ng/mL phorbol-12-myristate 13-acetate (PMA, Sigma-Aldrich, Saint Luis, MO, USA, #P8139) at a concentration of 5 × 10^5^ cells/mL in 12-well plates or 2 × 10^6^ cells/mL in 6-well plates for 24 h for differentiation into macrophages. THP-1 cells were resuspended in fresh medium at a concentration of 2 × 10^5^ cells/mL in 24-well plates or 1 × 10^6^ cells/mL in 6-well plates overnight. Cells were stimulated with or without IL-25 (2, 10, and 40 ng/mL) (PEPROTECH, East Windsor, NJ, USA, #200-24) at different time points.

### 4.2. Measurement of ROS Production

2′-7′-Dichlorofluorescein diacetate (DCFH-DA, Sigma-Aldrich, #D6883) was used to measure ROS production by flow cytometry.

The highly fluorescent compound 2′,7′-dichlorofluorescein (DCF) was oxidized by ROS from nonspecific esterase-cleaved DCFH-DA. THP-1 cells and THP-1 derived macrophages were incubated with 5 μM DCFH-DA at 37 °C for 10 min before IL-25 treatment or pretreated with N-acetylcysteine (NAC, #A7250), vitamin C (#A5960), antimycin A (#A8674), MitoTEMPO (#SML0737), AMPK inhibitor dorsomorphin (#P5499), or mdivi-1 (#M0199) (all from purchased Sigma-Aldrich) for 30 min. Antimycin A, a mitochondrial respiratory chain III inhibitor, interrupts the electron transfer from the cytochrome b to the Qi-site, disrupting the mitochondrial Q-cycle of enzyme turnover [50]. MitoTEMPO, a triphenylphosphonium derivative, is easier to pass through all biological membranes and accumulate within mitochondria than non-targeted parent antioxidants [51].

Then, cells were washed twice with PBS. As stated above, intracellular H_2_O_2_ or low-molecular-weight peroxides can oxidize DCFH-DA to the highly fluorescent compound DCF. Signals were detected with a 525 nm bandpass filter (FITC) using an LSR II flow cytometer (Becton Dickinson, San Jose, CA, USA).

### 4.3. Measurement of Mitochondrial Complex Activity

The mitochondrial fraction was isolated from THP-1 cells and THP-1 derived macrophages using a mitochondria isolation kit (#ab110170), followed by freeze/thaw steps (dipping the vial into liquid nitrogen for 1 min and then 37 °C until thawing) and centrifugation at a higher speed to isolate mitochondria. The activity of complex I in whole cell lysates of THP-1 cells or THP-1 derived macrophages was measured using a complex I enzyme activity microplate assay kit (#ab109721) by following the oxidation of NADH to NAD^+^ and the simultaneous reduction of a dye, which leads to increased absorbance. Complex II/III activity was measured in isolated mitochondria using a MitoTox™ complex II+ III OXPHOS activity assay kit (#ab109905). Succinate and oxidized cytochrome c were added to the mitochondria. The rate of the coupled complex II + III reactions was measured by monitoring the conversion of oxidized cytochrome c into reduced form, which can be observed an increase in absorbance. All assay kits including mitochondria isolation kit were purchased from Abcam (Abcam, Cambridge, MA, USA).

### 4.4. Western Blotting

After 8 h of IL-25 treatment or pretreatment with the NAC, rotenone (Sigma-Aldrich, #R8875), antimycin A, AMPK, or Mdivi-1, THP-1 cells and THP-1 derived macrophages were lysed with equal volumes of ice-cold lysis buffer. Equal amounts of cell lysates were separated using SDS-PAGE and transferred to PVDF membranes. After blocking in PBS containing 5% milk and 0.1% Tween 20, the membrane was incubated with anti-phospho (p)-5′-AMP-activated protein kinase alpha (AMPKα, #2535), anti-AMPKα (#5832), anti-PINK1 (#6946), anti-GAPDH antibodies (#2118S) (all from purchased Cell Signaling Technology, Danvers, MA), anti-p-Parkin (Abcam, Cambridge, MA, #ab73015), anti-LC3 (GeneTex, CA, USA, #GTX127375), or anti-Parkin (Abcam, #ab77924) to further investigate the influence of IL-25 on mitophagy. Immunoreactive bands were visualized using a horseradish peroxidase-conjugated secondary antibody and an enhanced chemiluminescence (ECL) system (Merck Millipore, Darmstadt, Germany, #WBKLS0500). The assay was performed by using the protocols recommended by the manufacturer. After chemiluminescent detection of phosphoproteins, we used Gentle Review™ Stripping Buffer (VWR LIFE SCIENCE, Pennsylvania, USA, #N552) to strip the PVDF membrane. We added 10 mL stripping buffer to incubate the membrane with gentle sharking for 30 min at room temperature.

### 4.5. Confocal Immunofluorescence Microscopy

The non-adhesive THP-1 cells were cultured with PMA in 12 wells containing a coverslip and differentiated to adhesive cells, macrophages. After IL-25 treatment, THP-1 cells and THP-1 derived macrophages were stained with MitoTracker Deep Red (Invitrogen, Waltham, MA, USA, #M22426) 400 nM for 30 min at 37 °C. After MitoTracker staining, the THP-1 cells were collected in 1.5 mL eppendorf, and fixed with 4% paraformaldehyde, permeabilized with 0.2% Triton X-100, blocked with PBS containing 1% BSA, and finally incubated with the anti-LC3 antibody (1:100) in blocking buffer at 4 °C overnight with end-to-end rotation. After washing, the cells were incubated with a fluorescein-conjugated goat anti-rabbit antibody (1:100, Vector Laboratories Inc., Burlingame, CA, USA, #A11008) in a blocking buffer with end-to-end rotation. After another wash, the cells were stained with the nuclear stain 4′,6-diamidino-2-phenylindole (DAPI) (300 nM; Invitrogen, #D1306) in PBS. Finally, the cells were centrifuged at 1000 rpm for 5 min by Shandon Cytospin Cytocentrifuge (Thermo Fisher Scientific, Waltham, MA, USA), mounted with ProLong™ Gold antifade mounting medium (Invitrogen, #P36930), and covered with glass coverslips. After MitoTracker staining, THP-1 derived macrophage on a coverslip in 12 wells plate were stained following the step described above with an orbital shaker. After mounted, we imaged using an LSM 700 and a 63×/1.4 NA objective lens. (Carl Zeiss Microscopy, Göttingen, Germany).

### 4.6. Enzyme-Linked Immunosorbent Assay (ELISA)

After pretreatment with IL-25 for 2 h, THP-1 cells were cultured with 10 ng/mL PMA, PMA combined with 10 ng/mL human IFN-γ recombinant protein (PEPROTECH, #300-02) and 10 ng/mL lipopolysaccharides (Sigma-Aldrich, #L2880) or PMA combined with 10 ng/mL human IL-4 recombinant protein (PEPROTECH, #200-04) for differentiation into macrophages, M1 macrophages or M2 macrophages. The TNF-α (#DY210), CXCL-10 (#DY266), and CCL-22 (#DY336) concentrations of cell supernatants were collected at 48 h and measured using a commercial ELISA kit. (R & D Systems, Minneapolis, MN, USA). Mdivi-1, mitochondrial division inhibitor-1, is a selective inhibitor of Drp1 that leads to mitochondrial fission and mitophagy [52,53]. The CCL-22 concentration in the supernatant of IL-25-treated macrophages and M2 macrophages treated with or without Mdivi-1 was measured. CCL-22 concentrations in THP-1 and PMA/IL-4-primed THP-1 cells treated with or without the mitophagy inhibitor Mdivi-1 were detected by ELISA.

### 4.7. PINK1 Knockdown

THP-1 cells were transduced with multiplicity of infection (MOI) 1 of shRNA lentiviral particles encoding either a non-targeting (nt) shRNA (#TRCN0000208001) or shRNA targeting PINK1 (#TRCN0000007097 (targeting in 3′ UTR), #TRCN0000007101, and #TRCN0000199446 (both targeting in CDS)), and spin cells at 2250 rpm for 30 min at 37 ℃ according to the protocol of the RNAi core facility. In addition, THP-1 derived macrophages were also transduced with multiplicity of infection (MOI) 1 of shRNA lentiviral particles as described here above. A multiplicity of infection (MOI) is the number of transducing lentiviral particles per cell. All shRNA lentiviral particles were purchased from the RNA technology platform and gene manipulation core of Academia Sinica. According to the manual instruction of the RNAi core facility, the cells that transduced with more than MOI 3 would have had an off-target effect according to the analysis of RNA sequencing. In this study, we transduced shRNA lentiviral particles to THP-1 cells only with MOI 1 condition, which could almost avoid the off-target effect resulted from gene knockdown by RNA interference. For the selection of successfully transduced cells, puromycin (final concentration 0.5 µg/mL) was used. After 3 d, PINK1 protein expression in PINK1 shRNA (#TRCN0000007097 and #TRCN0000199446)-transduced cells were both reduced by 40% compared to that in nt shRNA-transduced cells via Western blot. According to the outcome of knockdown efficiency by Western blotting, we used TRCN0000007097 and TRCN0000199446 for further investigation of the effects of IL-25 on ROS production, subsequent mitophagy, and CCL-22 concentration of M2 macrophages. PINK1 knockdown cells were pretreated with IL-25 for 2 h and differentiated into M2 macrophages. After 48 h, the supernatant was collected, and the CCL-22 concentration was measured.

### 4.8. Statistical Analysis

All data are presented as the means ± standard deviations. The Mann–Whitney U-test was used in each independent experiment to analyze the difference between the experimental and control groups. The densitometric data from Western blot analysis were analyzed using ImageJ software (National Institutes of Health, Bethesda, Maryland, USA) to measure the optical density of each band. The mean fluorescence intensity (MFI) of intracellular ROS level was analyzed using FCS Express 4 Flow Research (De Novo Software, Pasadena, USA) All data were analyzed using GraphPad Prism version 5.0 software (GraphPad Software Inc., San Diego, CA, USA) to determine differences between groups. A *p* value < 0.05 was considered to indicate a significant difference.

## Data Availability

The data presented in this study are available on request from the corresponding author. Data may be available upon request to interested researchers. Please send data requests to Chih-Hsing Hung, MD, PhD. Department of Pediatrics, Kaohsiung Medical University Hospital, Kaohsiung Medical University.
